# An Empirical Analysis of Primary and Secondary Pharmaceutical Patents in Chile

**DOI:** 10.1371/journal.pone.0124257

**Published:** 2015-04-27

**Authors:** María José Abud, Bronwyn Hall, Christian Helmers

**Affiliations:** 1 School of International and Public Affairs, Columbia University, New York, United States of America; 2 Department of Economics, University of California, Berkeley, California, United States of America; 3 UNU-MERIT, University of Maastricht, Maastricht, The Netherlands; 4 Department of Economics, Santa Clara University, Santa Clara, California, United States of America; University of Minnesota, UNITED STATES

## Abstract

We analyze the patent filing strategies of foreign pharmaceutical companies in Chile distinguishing between “primary” (active ingredient) and “secondary” patents (patents on modified compounds, formulations, dosages, particular medical uses, etc.). There is prior evidence that secondary patents are used by pharmaceutical originator companies in the U.S. and Europe to extend patent protection on drugs in length and breadth. Using a novel dataset that comprises all drugs registered in Chile between 1991 and 2010 as well as the corresponding patents and trademarks, we find evidence that foreign originator companies pursue similar strategies in Chile. We find a primary to secondary patents ratio of 1:4 at the drug-level, which is comparable to the available evidence for Europe; most secondary patents are filed over several years following the original primary patent and after the protected active ingredient has obtained market approval in Chile. This points toward effective patent term extensions through secondary patents. Secondary patents dominate “older” therapeutic classes like anti-ulcer and anti-depressants. In contrast, newer areas like anti-virals and anti-neoplastics (anti-cancer) have a much larger share of primary patents.

## Introduction

Historically, pharmaceutical patents are among the most controversially debated issues with regard to intellectual property (IP) protection, especially in developing countries. During the negotiations of the Trade-Related Aspects of Intellectual Property Rights (TRIPS) Agreement, pharmaceutical product patents represented one of the most divisive issues, being opposed by developing countries because of concerns that stronger patent protection would hinder access to drugs and prevent the development of a domestic pharmaceutical industry. The TRIPS agreement forced developing country members of the World Trade Organization (WTO) to grant patents with a statutory lifetime of 20 years from the patent application also to pharmaceutical compounds. Almost two decades after TRIPS, the empirical evidence on its effect on developing countries is at best mixed [1–3].

Despite the strengthening of IP protection brought about by TRIPS, some developing countries continue to apply a more restrictive approach than developed countries to the granting of pharmaceutical patents. While TRIPS requires the availability of patent protection for processes as well as products in “all fields of technology” (TRIPS Article 27.1), the agreement provides countries with substantial freedom to define the standards of patentability. Some developing countries, most prominently India (Section 3(d) of India’s Amended Patents Act of 2005), have used this freedom to restrict the granting of so-called secondary pharmaceutical patents. As opposed to primary patents which protect an active ingredient directly, secondary patents protect a range of chemicals related to an active ingredient (such as crystalline forms of the original compound), methods of use, formulations, dosages, etc. Other developing countries, such as Brazil, Argentina and South Africa, are currently debating new legislation that would emulate India’s approach to restricting the patentability of secondary patents.

In developing countries, secondary patents may have played particularly important a role for multinational originator companies during the years following the introduction of pharmaceutical patents. When developing countries began to allow the granting of pharmaceutical product patents, in many instances originator companies were unable to obtain patent protection for drugs that had already been patented abroad. In Chile, for example, pharmaceutical patents were introduced in 1991, but pharmaceutical drugs that had been patented abroad before the 1991 law came into effect were expressly not patentable. This may have created strong incentives for originator companies to rely on new secondary patents instead.

The sparse, available evidence on secondary patents, which focuses on the U.S. and the European Union (EU) (see the next section), offers some evidence on the use of secondary patents by originator companies. Empirical and anecdotal evidence suggests that pharmaceutical originator companies use secondary patents extensively in those markets. There is also some evidence that secondary patents can be used to extend patent protection on a given drug in length and breadth and it may create legal uncertainty over the scope of patent protection of a drug. That said, secondary patents can be used to protect genuine follow-on innovation, although distinguishing strategic use of secondary patents from their use to protect follow-on innovation is very difficult and may not even be feasible when such patents serve both purposes.

Despite the widespread use of secondary patents and the contentious policy debate, there is little evidence on the use of primary and secondary patents in developing countries. Our objective in this paper is to shed light on the use of primary and secondary patents by multinational originator companies in Chile and to gauge their effect on creating and maintaining exclusivity.

From a data point of view, studying this question is challenging because it requires not only a distinction between primary and secondary patents, but also a mapping of patents to active ingredients and the corresponding pharmaceutical products. Linking patents to active ingredients is an enormous challenge because there is usually no explicit mention in the patent claims of the active ingredient contained by a drug (where drugs can contain multiple active ingredients). We create a new dataset that addresses this problem in three ways. First, we rely on the Orange Book of the U.S. Food and Drug Administration (USFDA) to identify U.S. patents on the compounds registered in Chile. We then construct patent families for these U.S. patents and verify whether there are any Chilean equivalents. Similarly, we undertake the same exercise using the Merck Index, which provides information on patents worldwide. Second, we use a dataset created by the Chilean patent office (INAPI) that contains the compound-patent mapping for all new compounds registered in Chile between 2005 and 2010. All of these matches are based on patent applications, whether granted or not. Third, we asked experts in pharmaceutical patents in Chile to match directly the remaining set of all *granted* Chilean patents to the complete list of drugs registered with the Chilean health authorities. This means that we attempted to match all granted Chilean patents to drugs, either directly or through any of the other approaches, although most of the patents do not match, as we show below. Unfortunately, for cost reasons, we were unable to search the remaining patent applications for matches to registered drugs, but based on our earlier match rates, we expect there to be very few of these.

Because companies can obtain competitive advantage also through brand recognition, we also match the pharmaceutical product-level data with trademark data. The mapping of drugs and trademarks is more straightforward than that of drugs and patents. The pharmaceutical product data provides the names under which drugs are marketed, which we search for in the relevant classes in our trademark database.

For the matching of patents and trademarks, we rely on a dataset that contains the universe of patent and trademark applications filed with the Chilean patent office (INAPI) between 1991 and 2010 [4]. The pharmaceutical product data comes from the National Public Health institute (ISP). In Chile, all pharmaceutical products that are to be sold on the domestic market have to be registered with the institute. It maintains a database that links all registered drugs in Chile to the pharmaceutical compounds that they contain.

Our study contributes to the sparse empirical literature on the use of secondary patents, in particular by foreign multinationals. It offers in particular for the first time empirical evidence on the use of secondary patents in a developing country.

## Primary and Secondary Patents

In the pharmaceutical industry, patents are usually filed already during the research phase in the development of a new drug. These early patents are filed to protect potential active ingredients that form the basis of the new drug. Since the early stages of drug development are characterized by an enormous amount of uncertainty (the European Commission [5] suggests that 1 in 5,000–10,000 test active ingredients results in a successful drug), early patent filings reflect this, in that many of these filings will either not be pursued, or if granted, will never be related to a marketed drug. Patents on active ingredients are referred to as primary patents. In later phases of the drug development, patents are filed on other aspects of active ingredients such as different dosage forms, formulations, production methods etc. These patents are referred to as secondary patents. Secondary patents also emerge from changes to formulations and dosages or applications in new therapeutic classes, discovered during clinical trials. [6] reports that the usual filing strategy is to file many and broad primary patent applications and then to surround them with secondary patent applications.

A critical issue regarding secondary patents is whether they protect genuine follow-on innovation or whether they represent primarily a form of strategic patenting (although these two may not necessarily be mutually exclusive). There is little controversy about the innovation associated with new active ingredients. However, new uses of existing active ingredients in new therapeutic areas, new formulations, new modes of delivery, new combinations of known active ingredients etc. are sometimes regarded as incremental innovation. In this case, secondary patents represent a way of incentivizing and protecting potentially valuable follow-on innovation. This may be particularly valuable for generics producers that want to develop proprietary drugs by modifying existing active ingredients as a lower risk strategy. For example, consider a new formulation that allows administering an active ingredient in form of a temperature-stable pill instead of a temperature-sensitive soft-gel version [7]. It is clear that the pill has no added therapeutic benefit over the soft-gel version; at the same time the pill represents an improvement over the soft-gel in terms of ease of drug storage and administration. On the other hand, secondary patents may also be used to extend the time of market exclusivity and to maintain or even expand the market that the product covers during market exclusivity. These objectives can be supported by specific patenting strategies, in particular the creation of patent fences and clusters. According to [8], “a key element of any life cycle management strategy is to extend patent protection beyond the basic patent term for as long as possible by filing secondary patents which are effective to keep generics off the market.”

The scarce available evidence on secondary patents suggests that secondary patents are pervasive and that they seem to be used overwhelmingly as a strategic tool. For example, the European Commission found in its 2009 pharmaceutical sector inquiry a primary to secondary patent ratio of 1:7 [5]. This ratio is higher for pending than granted patents (1:13 vs. 1:5), which suggests that a large number of secondary patent filings are not granted, presumably because they do not meet the statutory patentability requirements or because they are not pursued by the applicant, having served their purpose of increasing uncertainty. The inquiry shows that 57% of secondary patent filings protect formulations, 7% devices, 7% combinations of known active ingredients, 5% polymorphic forms, 4% salts, and the remaining 20% are accounted by a range of claims, such as hydrates or solvates [5]. The study also reveals that if the validity of an originator’s patent is challenged either through post-grant opposition or an invalidation action in court, the majority of secondary patents is invalidated as a result (or their claims restricted) [6]. [9] conduct a similar study for the U.S. They look specifically at patenting associated with 342 new active ingredients approved by the U.S. FDA between 1991 and 2005. They find that around 50% of drugs are protected by secondary patents. There is an increase in the share of drugs with secondary patents over time whereas the share of drugs protected by primary patents remains constant. This filing pattern was also found by [10] who studies the patenting behavior of companies that market Phosphodiesterase Type 5 inhibitors (for the treatment of erectile dysfunction). He also finds that the originator companies included in his study, Pfizer, Bayer and Ely Lilly, file a large amount of secondary patents during later stages of the life-cycle of a drug. This is suggestive of the fact that secondary patents are filed later in the life cycle of a drug to extend the patent life. In fact, the data for the U.S. by [9] reveals that compound patents are filed before FDA approval whereas secondary patents are filed mostly after approval. The authors estimate that secondary patents generate between 4–5 years of additional patent life on top of compound patents associated with a drug. The mean masks considerable variation. For example, [7] found for their case study of two HIV drugs that secondary patents extend patent protection up to 12 years beyond the lifetime of the original primary patents. Although this number might overstate the effective extension of a drug’s patent protection because [7] include patent applications (as opposed to grants) as well as granted patents not listed in the Orange Book (which may be a lot less effective in preventing generic entry). Another example is Sanofi Aventis’s ARAVA arthritis drug in Australia. Sanofi Aventis effectively extended exclusivity by 10 years through secondary patents (Sanofi-Aventis Australia Pty Ltd v Apotex Pty Ltd (No.3) [2011] FCA 346). Other examples of blockbuster drugs are GlaxoSmithKline’s antidepressant Paxil or Pfizer’s cholesterol-lowering Lipitor. In both cases, secondary patents extend patent protection by several years relative to the original compound patents [6,11]. It is, therefore, not surprising that the available evidence indicates a positive correlation between the number of secondary patents for a given drug and higher sales.

An important element in the filing strategy of secondary patents is the creation of legal uncertainty. For example, in their study of HIV drugs [7] found overlapping patent claims for a number of formulation patents. They also show that some of the formulation patents protect variations of known excipients (for example on new flavors such as peppermint or vanilla), or combinations of known excipients. According to their assessment, these patents are likely invalid. [8] report the case of AstraZeneca’s Prilosec. While courts in the U.S. upheld secondary patents that AstraZeneca had filed to extend the time of patent protection on Prilosec, the Patents Court in the U.K. invalidated the same formulation patents. This case illustrates that the question of validity of granted secondary patents is particularly unclear. [12] even conclude from their analysis of patent challenges by generics companies in the U.S. that challenges target secondary patents and are thus mostly used to restrain attempts by originator companies to extend patent terms beyond the original active ingredient patents through secondary patents.

## Regulatory Framework

### Registration of pharmaceutical products

Any drug marketed in Chile has to be registered with the Public Health Institute (ISP) – a government agency (Decree of the Health Ministry No. 1876 from 1995). The same rules apply regardless of whether the drug is imported or locally produced. Registration of new drugs with the ISP takes on average between 6 and 18 months. Registration fees are moderate (around US$2,300) and registrations have to be renewed after five years.

If a drug has already been registered on the ISP register, a company that wants to register a generic version can rely on the studies submitted for the first registration as proof of safety and efficacy provided the period of data exclusivity has expired. Also, since July 2008 (Resolution No. 3225/08), the ISP started requiring proof of bioequivalence for products that contain certain active ingredients. The number of affected active ingredients remained small during the period that we study (up to 2010), but has increased substantially since 2011 (see http://www.ispch.cl/medicamentos-bioequivalentes). For these products, the second party to register a drug has to submit studies of bioequivalence. However, because most drugs are still exempt from proving bioequivalence, most generics do not necessarily satisfy bioequivalence despite being pharmaceutically equivalent.

Patent protection is irrelevant for registration at the ISP. In contrast to the U.S. FDA for example, in Chile patent information concerning a new drug is neither requested nor verified when marketing approval is granted.

Apart from patent protection, the regulatory system in Chile also offers additional means for achieving exclusivity for new drugs. Data related to the safety and efficacy of new chemical entities provided for approval of new chemical entities is granted five-year exclusivity since 2005 (see also below), in cases where protection is requested by the applicant and granted by the ISP. This means that generics companies cannot refer to the data when applying for approval of a drug.

### Patents

Pharmaceutical drugs became patentable in Chile in 1991 through Law 19.039. The law offers patent protection for both products and processes and initially provided a statutory patent life of 15 years from the date the patent was granted, regardless of subject matter. The law excluded, however, all patents that had been applied for anywhere else in the world before the law came into force. Although the law still offered a way to obtain patent protection in Chile even if a patent had been granted in another jurisdiction before Law 19.039 entered into force (Law 19.039, Article 39), pharmaceutical patents were specifically exempted from this provision (Law 19.039 Transitional Provisions, Article 1).

Law 19.039 was amended several times during the period that we study (up to 2010): in 2005 by Law 19.996 and in 2007 by Law 20.160 (Law 17.336 in 2010 did not affect the patents contained in our sample.). The amendments brought Chile’s IP legal framework inline with TRIPS (taking advantage of the 10-year transition period for developing countries under TRIPS) and Chile’s obligations under FTAs with the U.S. and the European Free Trade Association (EFTA). Apart from a general extension of the patent term from 15 years from the date the patent was granted to 20 years from the application date, the most relevant changes affecting specifically pharmaceutical patents are the introduction of supplementary patent protection due to delays in the granting of a patent or the sanitary registration (Law 20.160, Article 53), the 5-year data exclusivity for new active ingredients mentioned above (Law 19.996, Article 89), a Bolar exemption (Law 20.160, Article 49), a softening of restrictions on second use patents (Law 19.996, Article 37e), and international exhaustion of patent rights (Law 19.996, Article 49) which effectively legalized parallel imports as long as the products were marketed abroad by the patent holder (or with the patent holder’s consent).

Finally, Chile joined the PCT system in 2009, which facilitates the international filing of patents. Although Chile’s accession to the PCT is likely to have had some effect on patent filings by foreign pharmaceutical companies in Chile, the change occurred in June 2009, which means it does not affect patent filings observed in our dataset.

## Data Description

To construct a dataset that combines patents and trademarks at the product level, we rely on a dataset that contains the universe of patents and trademarks filed with the Chilean patent office since 1991. This includes all patent and trademark applications by domestic as well as foreign entities, regardless of whether or not they have been granted.

To map patents to pharmaceutical products, we use the pharmaceutical registration data available at the ISP. The institute maintains a database that links all registered drugs in Chile to the pharmaceutical compounds that they contain. The database also contains additional information on the drug (e.g. when it was registered), the owner of the drug, whether the drug is produced domestically or abroad. We use the bridge between compounds and drugs contained in ISP’s database to link patents and trademarks at the product-level. Patents are linked to active ingredients whereas trademarks are linked to drug names. The link between patents and drugs represents a challenge as there is usually no explicit mention of the specific compounds in patent claims. Patents use the IUPAC (International Union of Pure and Applied Chemistry) classification to identify compounds whereas drugs rely on WHO’s INN (International Nonproprietary Name). Although compounds are usually described by a Markush structure in the patent, the same structure comprises often many functionally equivalent active ingredients; only the combination of specific examples provided in the patent and the Markush structure reveals the specific active ingredient protected by the patent (for details see the online appendix available at http://bit.ly/ahh_pharma).

We address this problem in three ways. First, we use a dataset compiled by INAPI that contains the compound-patent mapping for all new compounds registered with the ISP between 2005 and 2010. The mapping was undertaken by patent examiners specialized in pharmaceutical patents. Second, for all other compounds, we rely on the Orange Book of the U.S. FDA to identify U.S. patents on the compounds registered in Chile. We then construct patent families for these U.S. patents and verify whether there are any Chilean equivalents. Similarly, we undertake the same exercise using the Merck Index, which provides information on patents worldwide. Third, we asked specialists in pharmaceutical patents in Chile to match the remaining set of granted Chilean patents (nearly 3,000 patents) to our list of ISP products directly. As noted earlier, this leaves a large number of Chilean patent applications that neither matched to the Orange Book or Merck Index nor were granted that we were unable to search, but we expect that the matches to drugs in this set of patents will be very small in number.

The mapping between drugs and trademarks is more straightforward as the ISP database provides the names under which drugs are marketed, which we use to search for these drug names in our trademark database. In addition to matching drug names, we also match the names of all companies in the ISP database with the trademark register. Especially in the case of generics companies, individual drugs may not be trademarked, but the name of the company – which presumably appears on the packaging – will be.

The online appendix describes the data construction in more detail and Table 1 gives a summary of our patent-trademark match to the ISP register. Of 12,116 unique products registered at the ISP, 3,709 match to at least one Chilean patent, whereas 9,273 match to at least one Chilean trademark. After cleaning and translation of the active ingredients (including some standardization of names), there are far fewer active ingredients than products, as one might have expected. Of the 2,630 distinct active ingredients (many of which are common chemical compounds, that is, generics – for example vitamins), 322 match to at least one Chilean patent (504 distinct patents) and 2,332 match at to at least one Chilean trademark (10,461 distinct trademarks). Overall 82 per cent of the products and 91 per cent of the active ingredients are associated with some form of IP protection, more often trademark than patent.

**Table 1:** Matching results.

* *	* *	*Matched to*		*Shares matched*	
* *	*Total*	*patents*	*trademarks*	*patents*	*trademarks*
Unique ISP registrations	14,504	4,304	9,695	29.7%	66.8%
Unique product names	12,116	3,709	9,273	30.6%	76.5%
Unique active ingredients	2,630	322	2,332	12.2%	88.7%

**Fig 1:**
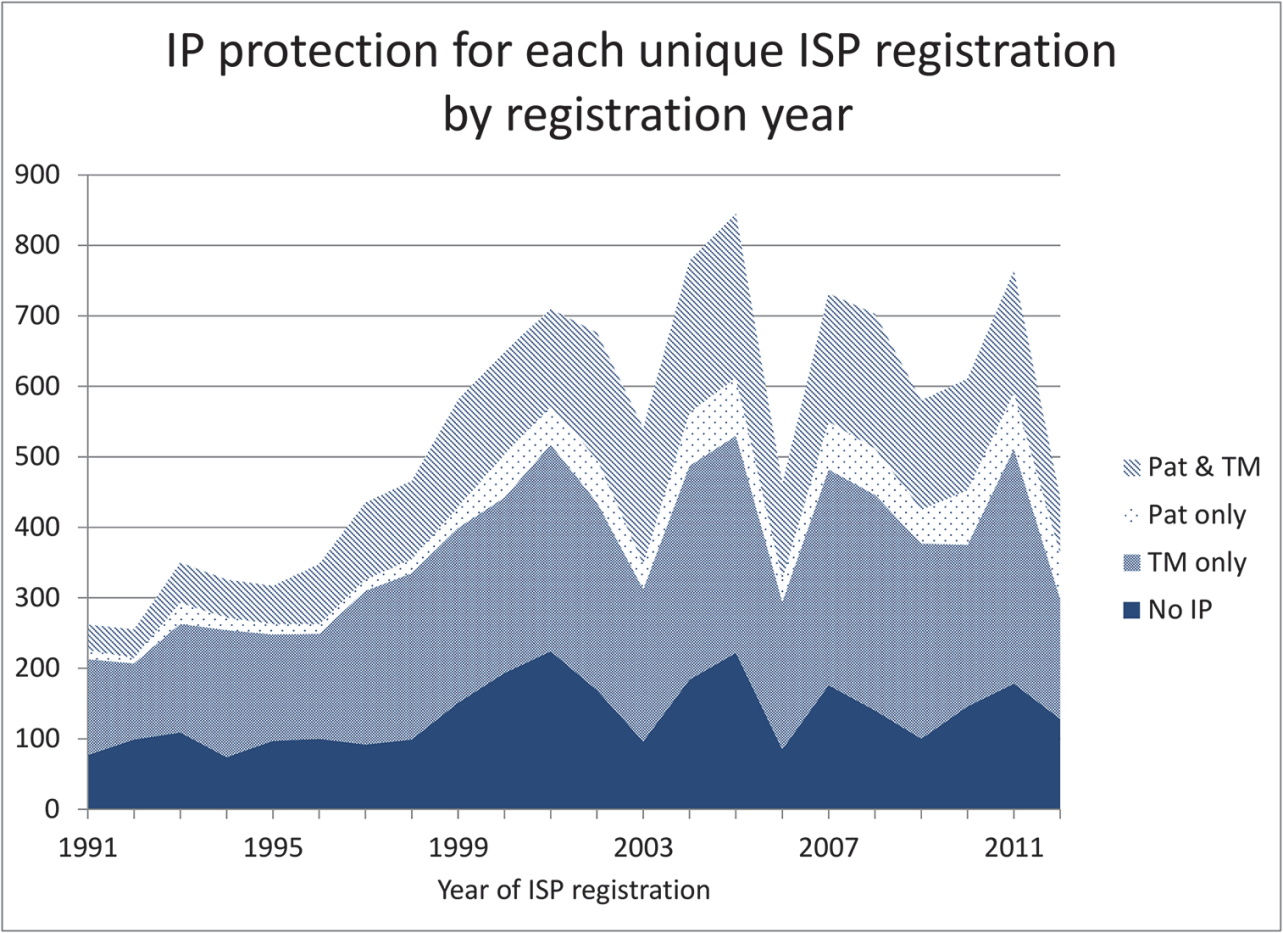
Time-trend patents and trademarks for unique product-active ingredient combination.

Fig 1 shows the time trends for the unique product-active ingredient combinations. There is a marked increase in the share using patents during the mid-1990s. Also the share relying only on trademarks increases substantially beginning the second half of the 1990s.

**Fig 2:**
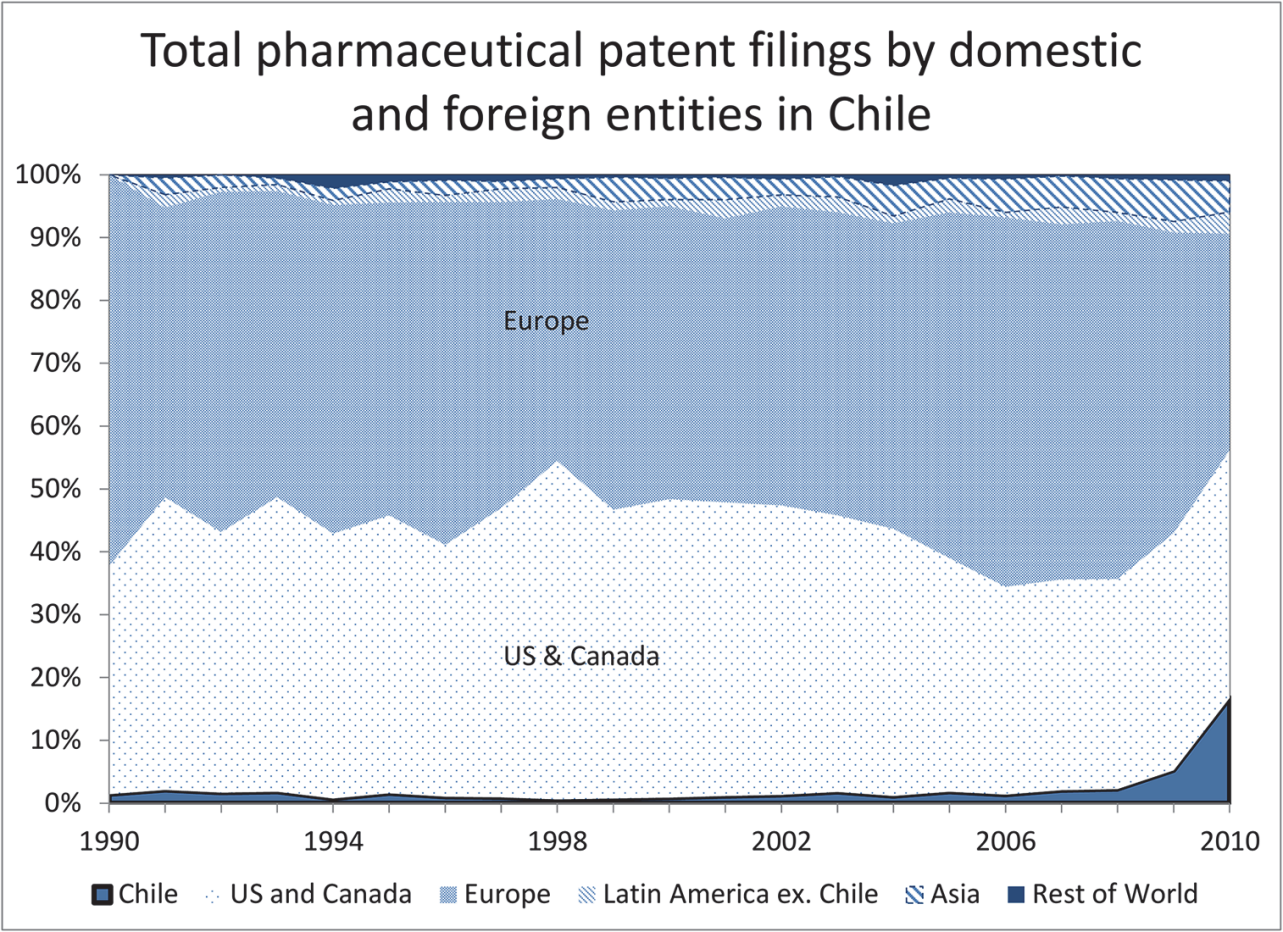
Time-trend patent filings by foreign and domestic entities.

**Fig 3:**
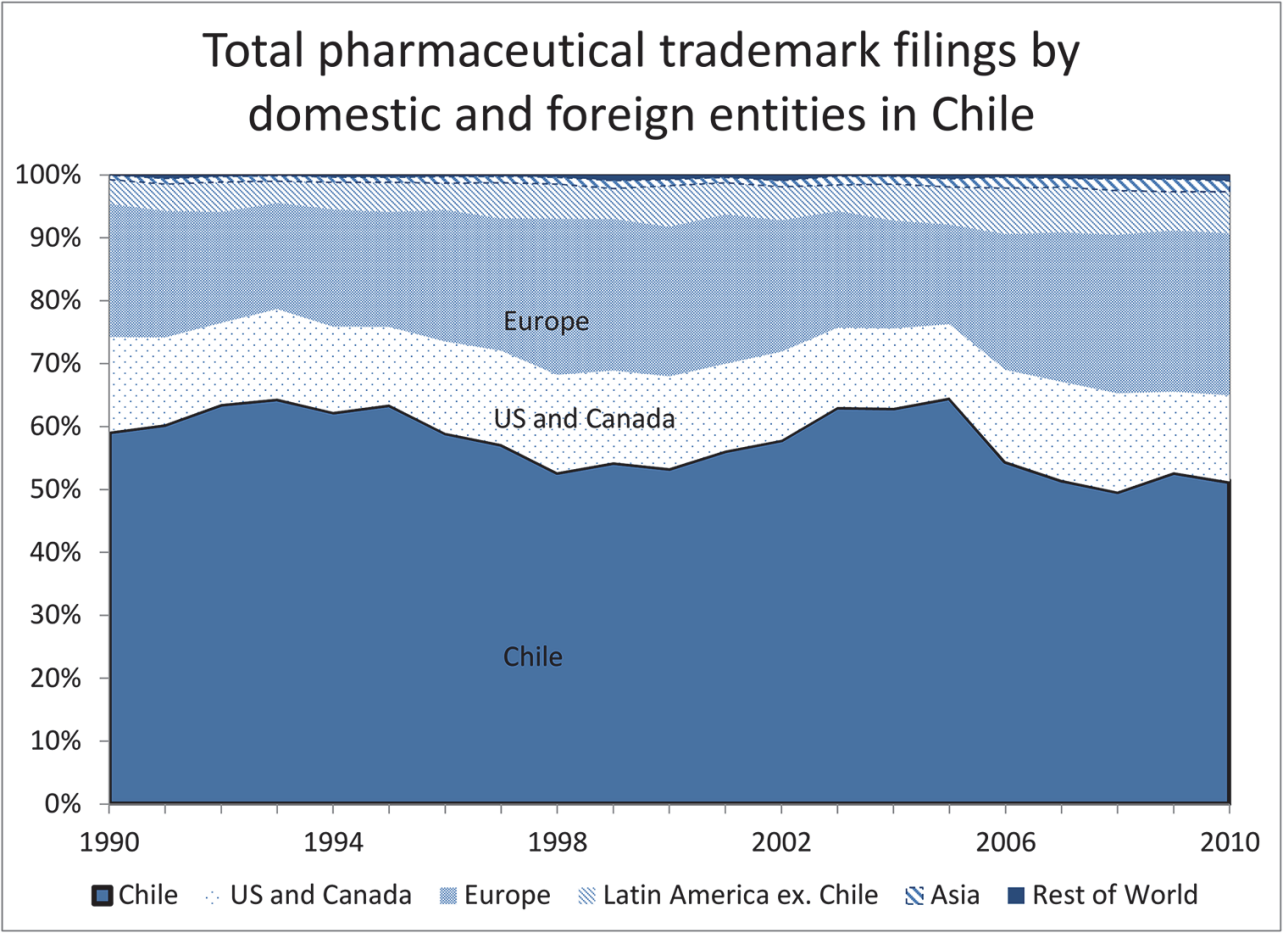
Time-trend trademark filings by foreign and domestic entities.

When we examine the ownership of this IP, we see striking differences in the regional patterns. Figs 2 and 3 show the share of patent (Fig 2) and trademark (Fig 3) filings coming from domestic and foreign entities in Chile, by date of the corresponding ISP registration. Almost all the patent filings are by entities based in Europe and the U.S., with the exception of a small increase in Chilean-origin filings during the most recent period. The total share of Chilean-origin filings is less than two per cent of total pharmaceutical patent filings, and none of these filings match to active ingredients in the ISP registration data. In contrast, over half the trademark filings are by Chilean entities, with the other half largely from Europe and the U.S. This pattern points to important differences in terms of the type of drugs marketed by domestic and foreign entities. Foreign originator companies rely on exclusivity through patent protection (in combination with trademark protection as shown in Table 2 below) whereas domestic companies compete (at least to some degree) through brand recognition in the generics market.

**Table 2:** Type of IP protection and primary/secondary patents.

*IP type*	*Active ingredient protected by primary patent only*		*Active ingredient protected by secondary patent only*		*Active ingredient protected by primary and secondary*	
	#	%	#	%	#	%
Patent only	3	5%	11	5%	1	3%
Patent and trademark	58	95%	210	95%	39	98%
Total	61		221		40	

## Results

Our main interest is in the use of secondary patents by foreign originator companies in Chile. Collecting the relevant data for investigating this question is challenging. We rely on the identification of our patents as primary or secondary that was done by internal and external patent examiners at INAPI following the classification proposed by [9]. Of the 504 Chilean pharmaceutical patents that match to our list of active ingredients, 113 (22%) were identified as primary patents, with the remaining 78% being secondary. This ratio of 1:4 is comparable to the ratio of 1:5 found for granted patents by the pharmaceutical sector inquiry of the European Commission [5]. If we look at all granted patents regardless of whether they have matched to a product registered at the ISP, we find that there are more primary than secondary patents. This could simply be the result of secondary patents facing a higher likelihood of rejection by the Chilean patent office which would be consistent with the findings of the EU Commission discussed above. Hence this does not necessarily mean that the ratio of primary to secondary patents is the same for patent applications (pre-grant).

The 504 matched patents are associated with 322 of the 2,630 active ingredients. Of these active ingredients, less than one third (101) have at least one primary patent. In about 88% of the cases with a primary patent, a primary patent is the first patent on that ingredient; in the remaining cases, there is a secondary patent preceding the primary patent.

**Fig 4:**
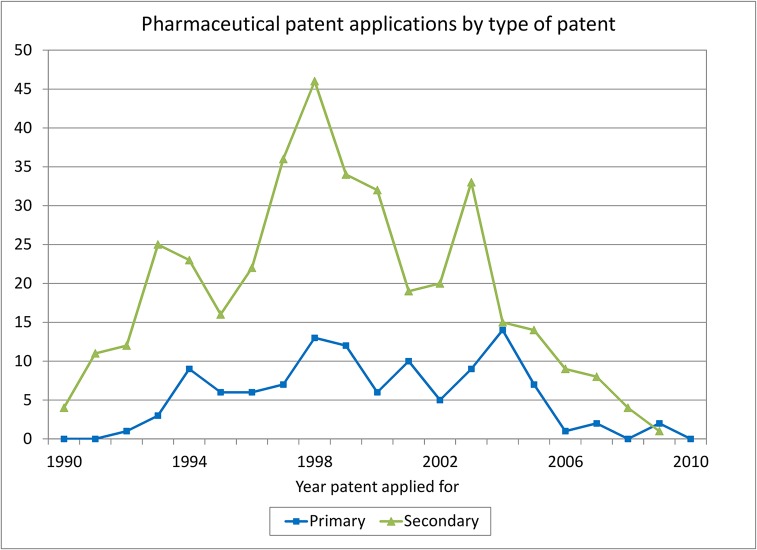
Pharmaceutical patent applications by type.

**Fig 5:**
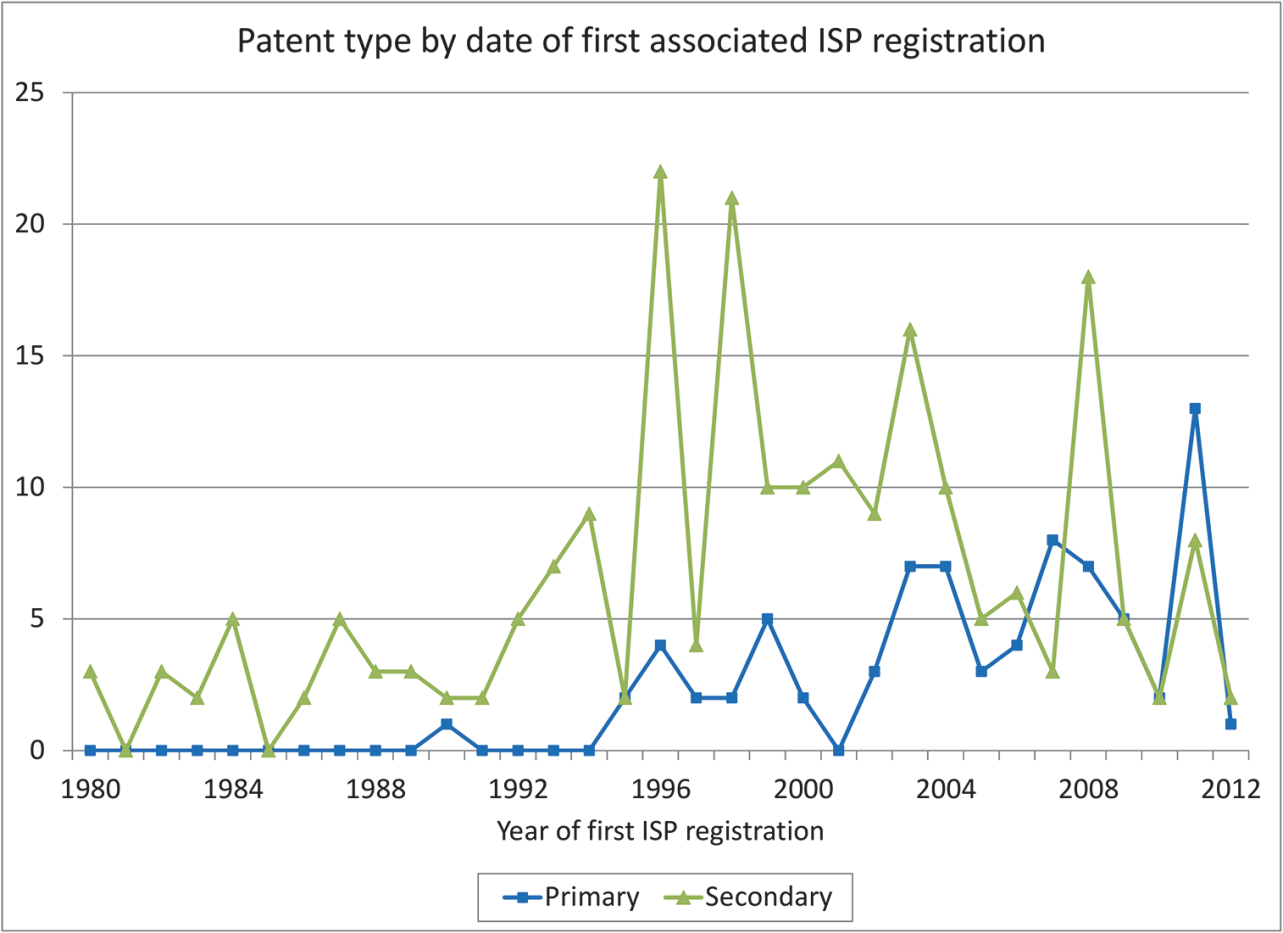
Pharmaceutical patent applications by year of first associated ISP registration.

**Fig 6:**
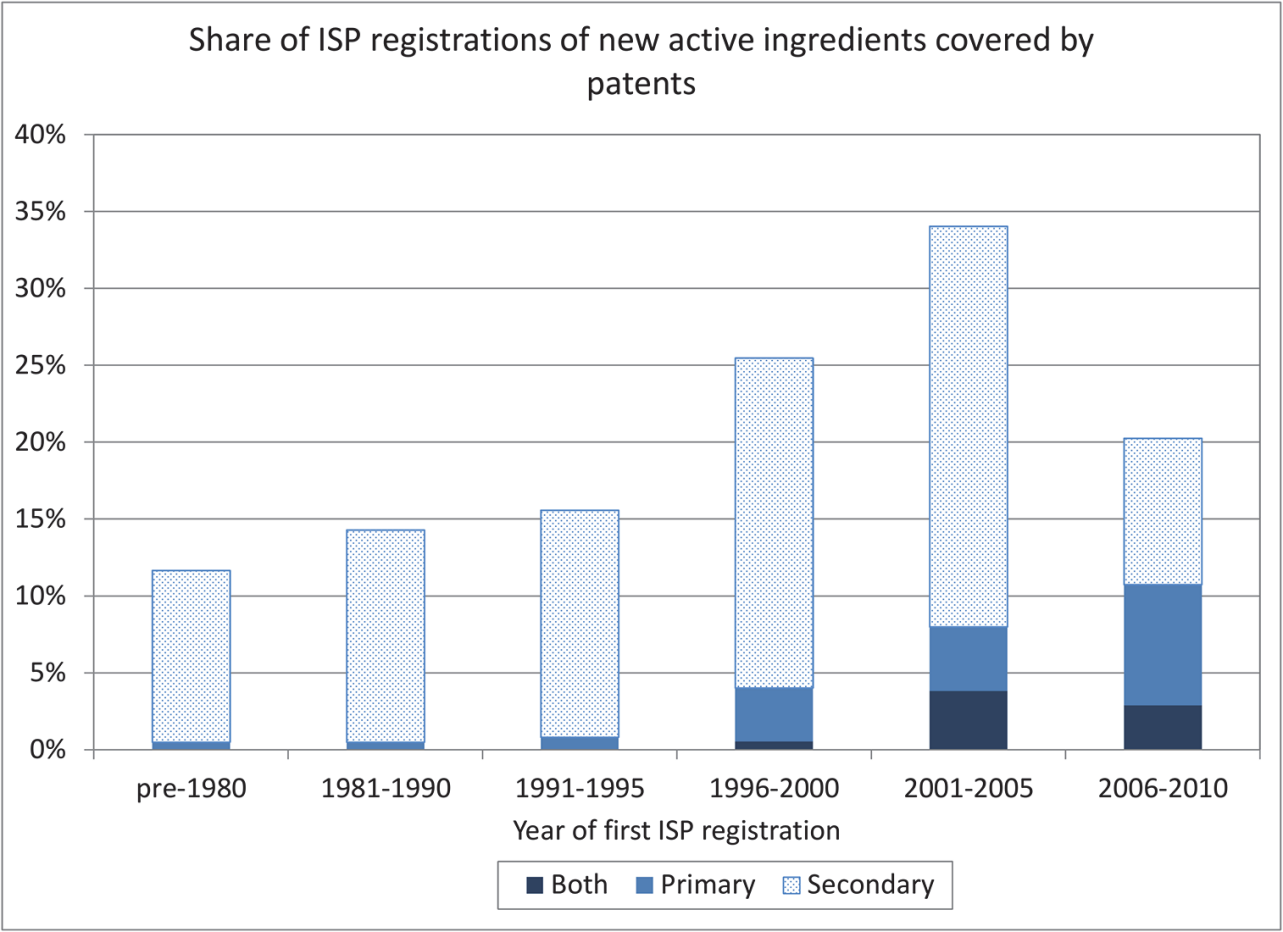
New ISP registrations and patenting.

Figs 4, 5 and 6 examine the patent-active ingredient match more closely. Fig 4 shows the trends in ISP-matched pharmaceutical patent applications for the two types of patents separately for the 1991–2010 period, by date of patent application. During the 1990s after the introduction of pharmaceutical product patents, both types of applications increase but after 2005 there is substantial decline, which may reflect the introduction of data protection and the worldwide slowdown in the introduction of new pharmaceuticals.

Figs 5 and 6 focus only on the ISP registrations that contain a new active ingredient and use the date of the ISP registration rather than the date of the patent application. Fig 5 counts unique patent applications, showing the breakdown between primary and secondary patents by the date of the first ISP registration containing an active ingredient that has been associated with the patent. There are 316 such patents; the remaining patents are associated with later appearances of the same active ingredient, because they are on compounds rather than single chemicals. Clearly there are almost no primary patents associated with pre-1991 ISP registrations, as one would expect given the absence of pharmaceutical product patentability. There are a number of secondary patents, however, suggesting that new formulations or uses of older ISP-registered products were patented after 1991.

Fig 6 also counts only unique ISP registrations the first time an active ingredient appears. It shows the share of these registrations that are covered by primary, secondary, or both types of patents during six time periods. This figure also makes it clear that patent coverage of new drugs is increasing, and that an increasing number of these drugs are covered by primary patents. Note that there may be some truncation during the 2006–2010 period due to incomplete patent data (patent applications that follow the ISP registration in the later years will be missing, implying an undercount of secondary patents, in particular). In spite of the increase in patent coverage, it is still the case that several hundred active ingredients registered at the ISP for the first time after the year 2000 are not associated with any Chilean patent applications. Many but not all of these ingredients are new virus vaccines or new ingredients for over-the-counter preparations such as vitamin compounds, etc.

**Fig 7:**
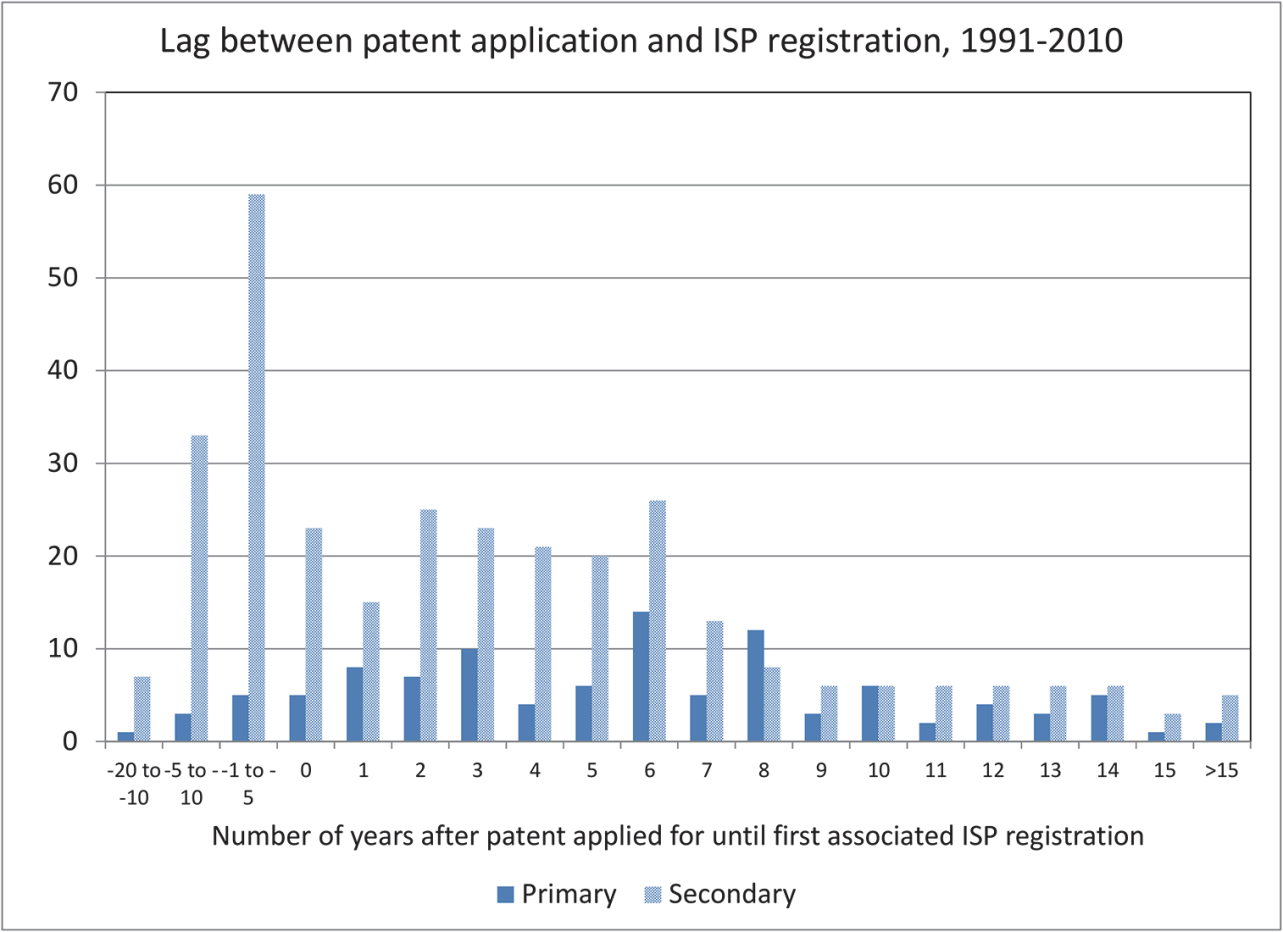
Lag between patent application and product registration.

To investigate the timing between a Chilean patent application and the first associated ISP registration further, we computed the lag between the two and plotted the distributions for primary and secondary patents in Fig 7. This figure clearly shows that the great majority (86%) of the primary patents are applied for before the first time the associated ingredient is registered at the ISP. In contrast, only 56% of the secondary patents are applied for before the initial ISP registration. A nonparametric test of the difference between the two lag distributions yields a χ^2^(1) of 37.5 and is highly significant. The median lag for primary patents is 6 years and for secondary patents it is 2 years. In a number of cases, the lags are over 5 years, which suggests delayed entry into the market.

**Fig 8:**
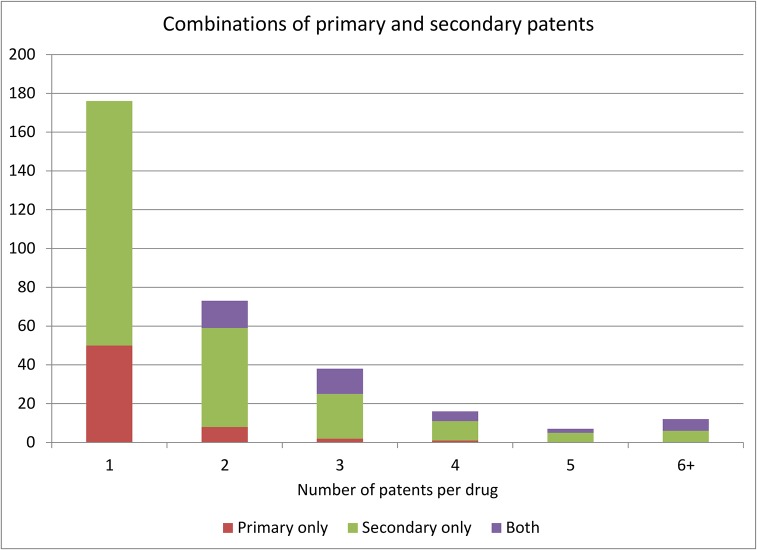
Combinations of primary and secondary patents.

Fig 8 looks at the number of patents that protect a given active ingredient. About 55% of the active ingredients are protected by a single patent and 34% of active ingredients that are patent protected are protected by 2 or 3 patents. Very few active ingredients are associated with a larger number of patents. When we look at the breakdown into primary and secondary patents 72% of active ingredients that are protected by a single patent are in fact protected by a secondary patent. Among drugs that are protected by several patents, in most cases they are protected by only secondary patents or a combination of primary and secondary patents.

Table 2 combines the data on trademark protection with the information on primary/secondary patents. This provides additional insight into the IP strategies of foreign originator companies. The table shows that nearly all active ingredients that are protected by a patent are also protected by a trademark. This indicates that companies rely on a patent-trademark combination to achieve market exclusivity. Moreover, there are no significant differences between active ingredients that are protected by either primary or secondary patents. That said, it appears that active ingredients protected by a combination of primary and secondary patents are even more likely to rely on both trademark and patent protection.

**Fig 9:**
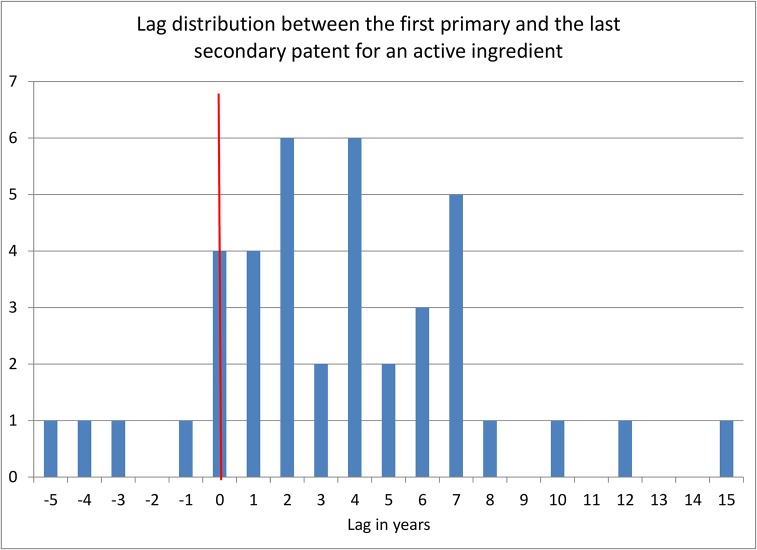
Lag between earliest primary patent and latest secondary patent by active ingredient (active ingredients protected by both primary and secondary patents).

To gauge the effect of secondary patents on potential patent term extensions, Fig 9 looks at the lag between the application date of the first primary patent and that of the latest secondary patent by active ingredient. The figure shows that in most cases the lag is positive, meaning the application for the secondary patent was filed after the primary patent, and in many cases this lag amounts to several years. If the secondary patent offered exclusivity to some degree, Fig 9 would suggest that in some cases, companies could gain a number of additional years of patent exclusivity through the filing of secondary patents. The median number of possible additional years is four, which is consistent with the numbers estimated by [9] for the United States. The active ingredient with the longest lag (15 years) is Posaconazole, an anti-fungal for which a crystalline form was patented in the United States 19 years after the original patent (see US patents 5278175 and 8435998).

Table 3 shows the number of primary and secondary patents associated with each therapeutic class (the total number of entries in the table is 1,246 because there can be more than one class for a given patent—see also Tables A-7-A-9 in the online appendix). The shares of primary patents vary considerably: recall that product patents were not available in Chile before 1991. This means that classes like anti-depressants and anti-ulcer (gastrointestinal agents) which had important patents prior to that date are covered largely by secondary patents. In contrast, newer areas like anti-virals and anti-neoplastics (anti-cancer) have a large share of primary patents.

**Table 3:** Number of patents per therapeutic class.

	*Number*		*Share*
*Therapeutic group*	*Primary patents*	*Secondary patents*	*Primary patents*
anti-viral agents	20	41	32.8%
anti-neoplastics	14	23	37.8%
anti-depressants	2	33	5.7%
anti-psychotics	1	31	3.1%
anti-diabetic agents	8	24	25.0%
analgesics	8	23	25.8%
nonsteroidal anti-inflammatory agents	7	20	25.9%
immunologic agents	9	13	40.9%
antibiotics/anti-neoplastics	5	17	22.7%
gastrointestinal agents (anti-ulcer)	2	19	9.5%
anti-fungals	3	16	15.8%
broncho-dilators	1	18	5.3%
anti-asthmatic combinations	3	15	16.7%
anti-histamines	2	15	11.8%
agents for pulmonary hypertension	1	15	6.3%
bone resorption inhibitors	0	16	0.0%
quinolones	3	12	20.0%
cholesterol absorption inhibitors	3	11	21.4%
hormones	1	11	8.3%
narcotic analgesics	2	10	16.7%
anti-infectives	2	10	16.7%
remaining classes	63	421	13.0%
**Total**	**160**	**814**	** **

**Fig 10:**
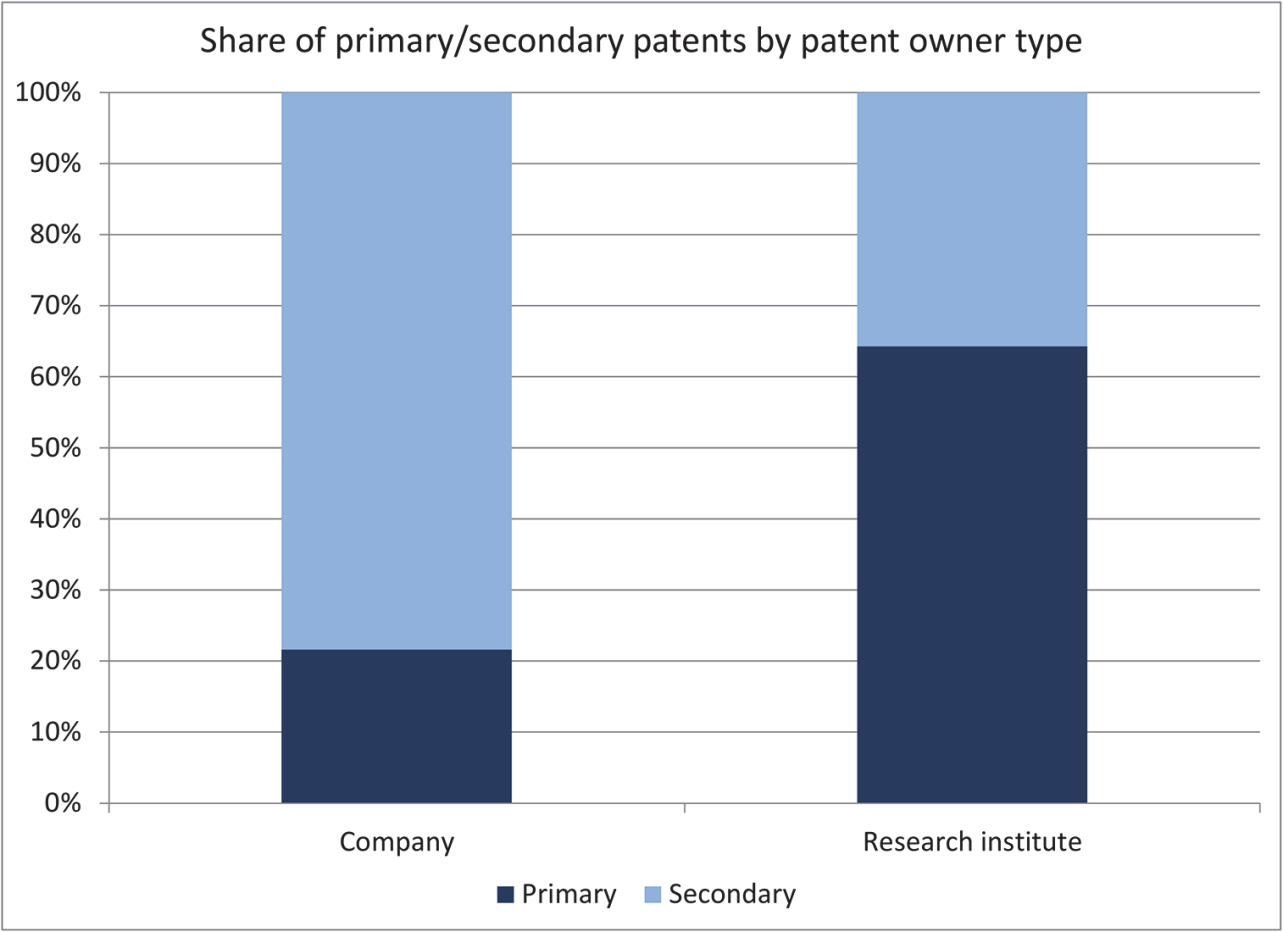
Share of primary/secondary patents by patent owner type.

One of way of assessing the importance of secondary patents for extending market exclusivity is to analyze whether there are any differences in the use of secondary patents by type of patent owner – in particular distinguishing between for-profit companies and not-for-profit research institutes and universities. In Fig 10 we distinguish between these two types of assignees. The figure shows that the share of secondary patents among patent-protected active ingredients is significantly larger for companies than for universities/not-for-profit research institutes. There are only 5 secondary patents that are assigned to universities and not-for-profit research institutes. However, with the exception of one secondary patent which is assigned to the Wellcome Foundation, all other secondary patents are co-assigned to universities/not-for-profit research institutes and private companies. This suggests that secondary patents are almost exclusively used by private companies as a tool to achieve exclusivity. That said, universities tend to focus on early stage research which is less likely to lead to the filing of secondary patents. Taking a closer look at patenting companies, we find that 76 out of 123 companies (62%) only file secondary patents whereas 25 companies (20%) only file primary patents (22 companies file both types of patents).

## Conclusion

Our objective was to take a first look at patenting of pharmaceuticals in Chile, with a particular focus on the distinction between primary and secondary patents. We provide a number of descriptive findings that show that pharmaceutical patents associated with drugs that have received market approval are almost exclusively the domain of foreign originator companies. Overall, we find that only a subset of drugs with market approval is protected by patents, a much larger number of products are protected by trademarks. We also find that foreign originator companies rely on a patent-trademark combination whereas domestic companies rely only on trademarks. Nevertheless, we also find a substantial number of ISP registrations that are not protected either by a patent or a trademark. When we take a closer look at ISP registrations protected by patents, we find that the majority are protected only by secondary patents (few active ingredients are protected by more than 1–3 patents). This is especially true before the change to the patent law in 1991, although it takes a few years for the number of primary patents to become significant. We also find that nearly all primary patents on active ingredients were filed before a drug containing the active ingredient was registered with the ISP. Secondary patents in contrast often follow with a lag of several years, that is, secondary patents are often filed after primary patents and after a drug has been registered at the ISP. The timing is also reflected in the fact that secondary patents dominate “older” therapeutic classes like anti-ulcer and anti-depressants. In contrast, newer areas like anti-virals and anti-neoplastics (anti-cancer) have a much larger share of primary patents. Our data also reveal that secondary patents are almost exclusively a tool used by private companies whereas universities and not-for-profit research institutes concentrate on primary patent protection.

This study is only a first step towards a better understanding of pharmaceutical patents in Chile. We have assembled a dataset that combines pharmaceutical products, active ingredients, patents, trademarks, and information on the corresponding companies. These data enable us to substantially deepen our understanding of the impact of patents on the pharmaceutical industry in Chile. Still, our approach and data have a number of obvious limitations. Perhaps most importantly, we only observe whether a drug has obtained market approval, but we have no information on actual demand or prices. This limits our ability to account for the importance of different drugs other than through their therapeutic classes.

We plan to extend this work to assess the impact that the combined use of primary and secondary patents has had on the ability of Chilean companies to compete in the generics industry. Such analysis could produce relevant insights for the current debate on secondary patents.
